# Enzymatic properties of alcohol dehydrogenase PedE_M.s. derived from *Methylopila* sp. M107 and its broad metal selectivity

**DOI:** 10.3389/fmicb.2023.1191436

**Published:** 2023-07-25

**Authors:** Ying Xiao, Kaijuan Wu, Syeda Sundas Batool, Qingqun Wang, Hao Chen, Xingyu Zhai, Zheng Yu, Jing Huang

**Affiliations:** ^1^Department of Microbiology, School of Basic Medical Science, Central South University, Changsha, Hunan, China; ^2^Department of Parasitology, School of Basic Medical Science, Central South University, Changsha, Hunan, China; ^3^Human Microbiome and Health Group, Department of Microbiology, School of Basic Medical Science, Central South University, Changsha, Hunan, China

**Keywords:** methylotrophs, lanthanides, pyrroloquinoline quinone, alcohol dehydrogenase, PedE_M.s.

## Abstract

As an important metabolic enzyme in methylotrophs, pyrroloquinoline quinone (PQQ)-dependent alcohol dehydrogenases play significant roles in the global carbon and nitrogen cycles. In this article, a calcium (Ca^2+^)-dependent alcohol dehydrogenase PedE_M.s., derived from the methylotroph *Methylopila* sp. M107 was inserted into the modified vector pCM80 and heterologously expressed in the host *Methylorubrum extorquens* AM1. Based on sequence analysis, PedE_M.s., a PQQ-dependent dehydrogenase belonging to a methanol/ethanol family, was successfully extracted and purified. Showing by biochemical results, its enzymatic activity was detected as 0.72 U/mg while the *K*_m_ value was 0.028 mM while employing ethanol as optimal substrate. The activity of PedE_M.s. could be enhanced by the presence of potassium (K^+^) and calcium (Ca^2+^), while acetonitrile and certain common detergents have been found to decrease the activity of PedE_M.s.. In addition, its optimum temperature and pH were 30°C and pH 9.0, respectively. Chiefly, as a type of Ca^2+^-dependent alcohol dehydrogenase, PedE_M.s. maintained 60–80% activity in the presence of 10 mM lanthanides and displayed high affinity for ethanol compared to other PedE-type enzymes. The 3D structure of PedE_M.s. was predicted by AlphaFold, and it had an 8-bladed propeller-like super-barrel. Meanwhile, we could speculate that PedE_M.s. contained the conserved residues Glu213, Asn300, and Asp350 through multiple sequence alignment by Clustal and ESpript. The analysis of enzymatic properties of PedE_M.s. enriches our knowledge of the methanol/ethanol family PQQ-dependent dehydrogenase. This study provides new ideas to broaden the application of alcohol dehydrogenase in alcohol concentration calculation, biosensor preparation, and other industries.

## Introduction

*Methylopila* sp. M107 is a member of methylotrophs from *α*-proteobacteria, which can use reduced substrates without carbon–carbon bonds as a carbon source (Chistoserdova, 2015). Owing to their unique metabolic system and enzymes, methylotrophs have been regarded as key metabolic bacteria since the 20th century. Their importance was proved by the research of Mary E Lidstrom (Puri et al., 2019), who constructed an artificial methane oxidation community by methylotrophs for the first time.They confirmed that methane oxidation was a stepwise oxidation process from methane to methanol to acetaldehyde to acetic acid to carbon dioxide, which is beneficial to promote the global carbon and nitrogen cycles as well as environmental protection. In particular, oxidation of alcohols is the most critical step in methane to carbon dioxide conversion pathway. Alcohol dehydrogenases (ADHs) are the main enzymes involved in this process, which oxidize alcohols to aldehydes or other salts and determine the type of assimilation pathway involved (Pfeifenschneider et al., 2017).

As an essential metabolic enzyme, ADH can catalyze the dehydrogenation of alcohols to aldehydes or ketones with a wide range of substrate specificities (Oppermann and Maser, 2000). According to the differences in the cofactors, ADHs can be divided into three main types: NAD^+^-dependent, oxygen-dependent, and PQQ-dependent ADHs (Aquino Neto et al., 2016), whereas ADHs in methylotrophic bacteria are mainly PQQ-dependent ADHs. PQQ-dependent ADHs are a large class of 8-bladed propeller-like dehydrogenases (Anthony and Williams, 2003). Compared to other types of ADHs, PQQ-dependent ADHs have received little attention. So far, most studies of PQQ-dependent ADHs have been performed in acetic acid bacteria (Trcek et al., 2006; Trček et al., 2007; Yakushi and Matsushita, 2010; Trček and Matsushita, 2013), these studies focused on correlation between acetic acid resistance and the characteristics of PQQ-dependent ADH. Moreover, it was discovered that PQQ can form a new coordination mode with Ca^2+^, Mg^2+^, La^3+^, Ce^3+^ and other metal ions (Toyama et al., 1997). The discoveries of a new coordination mode between PQQ and metal ions have shattered the understanding of the natural role of some metal ions, such as La^3+^. Therefore, the study of PQQ-dependent ADHs became attractive (Sarmiento-Pavía and Sosa-Torres, 2021). Mainly, redox centers of PQQ-dependent ADHs were cofactor PQQ and heme *c* moiety, which could oxidize substrates and undergo direct electron transfer with the electrode surface (Ivnitski et al., 2007; Ramanavicius and Ramanaviciene, 2009; Treu et al., 2009). Owing to the cofactors (such as metal ions) bound by PQQ and their tolerance to oxygen, PQQ-dependent ADHs are highly suitable for fabricating anodes in biosensors and biofuel cells (Yuhashi et al., 2005; Razumiene et al., 2006; Aquino Neto et al., 2016). Furthermore, PQQ-dependent ADHs also have a crucial physiological significance for plants (Choi et al., 2008) and mammals (including human) (Killgore et al., 1989). Although they have been widely found in eukaryotes (Matsumura et al., 2014; Takeda et al., 2015) and archaea (Sakuraba et al., 2010) such as methylotrophs, their functions or activities are still unknown.

A previous study reported that PQQ-dependent ADH is a tetramer of *α*_2_*β*_2_ and is divided into three main groups (Gvozdev et al., 2012). The first group is the *Type I quinoproteins* localized in the periplasm and containing a Ca^2+^-PQQ molecule (Anthony, 2001; Toyama et al., 2004) and *Type II quinohemoproteins* are localized in the periplasm and contain a Ca^2+^-PQQ and a covalently bound heme *c* moiety (Toyama et al., 2004; Gvozdev et al., 2012), while *Type III membrane-bound quinohemoproteins* are specific to acetobacter and consist mainly of dimeric or trimeric proteins on the cytoplasmic membrane. They are in close coordination and contact with each other. This study is centered on *Type II quinohemoproteins*, which are represented by Ca^2+^-dependent methanol dehydrogenases (MDHs) MxaF and ethanol dehydrogenases (EDHs) ExaA/PedE in prokaryotes. ExaA/PedE-type EDH is the key enzyme for methylotrophs, which allows microorganisms to obtain all the carbon and energy from ethanol or other reduced compounds lacking carbon–carbon single bonds for their survival and growth (Chistoserdova et al., 2009; Skovran and Martinez-Gomez, 2015; Chistoserdova and Kalyuzhnaya, 2018). Genome analysis of methylotrophs revealed that the ExaA/PedE-type alcohol oxidizing system involved at least 25 genes that participated in catalyzing and regulating alcohol (Keltjens et al., 2014). These genes are divided into five gene clusters *mxa*, *mxb*, *pqqABC/DE*, *pqqFG*, and *mxc*, which are closely related to the action and function of ADHs (Anderson and Lidstrom, 1988; Morris et al., 1995; Toyama et al., 1997).

In this study, we conducted the expression and purification of PedE_M.s. from *Methylopila* sp. M107. As shown by the biochemical results, PedE_M.s. had excellent enzymatic properties with ethanol as its optimal substrate. Moreover, PedE_M.s. was proved as a PedE-type EDH by the results that Ca^2+^ could improve its activity. Additionally, analysis of the predicted structure revealed that the α-subunits of PedE_M.s. consist of eight β-sheets, with the active center of PedE_M.s. being wrapped within (Sarmiento-Pavía and Sosa-Torres, 2021). Exploring the biochemical characteristics and structure of PedE_M.s. can significantly enhance our comprehension of the methanol/ethanol family PQQ-dependent dehydrogenase and provide insight into their physiological and biochemical basis for adapting to industrial production conditions. Thus, improving the industrial application value of ADHs is our aim.

## Materials and methods

### Bioinformatic analysis

Amino acid sequences with the first 100 similarities to PedE_M.s. were obtained using NCBI BLASTp.^1^ Clustal (Larkin et al., 2007) was used for amino acid multiple sequence alignments of PedE_M.s. and its homologs.^2^ In addition, ESpript (Robert and Gouet, 2014)^3^ was used to obtain secondary structure alignment results based on multiple sequence alignments. The phylogenetic tree was obtained by MEGA X using the neighbor-joining (NJ) method (Shirai et al., 2008).

### Gene synthesis and construction of expression vector

In this study, a modified pCM80 vector was utilized for protein expression, which has previously been employed successfully to express genes in *Methylorubrum extorquens* AM1 (Marx and Lidstrom, 2001). The protocols for constructing the modified vector were followed by the previous methods (Huang et al., 2019). The empirically derived codon usage table was used for the selected gene homologs which were codon-optimized for expression in *Methylorubrum extorquens* AM1. BOOST was used to perform codon optimization and vendor-defined synthesis constraint removal (Oberortner et al., 2017). Synthetic DNA was obtained from Twist Biosciences and was cloned into the *Nco*I site of the modified pCM80 vector using the Gibson Assembly method (NEBuilder HiFi, NEB). The *Escherichia coli* Top10 transformants were plated on lysogeny broth (LB) agar plates supplemented with tetracycline (10 μg/mL). Constructions of all genes were sequence-verified using the Tsingke Biotechnology sequencing (Changsha, China) platform.

### Plasmid transfer into the host strain

The quadruple mutant *Methylorubrum extorquens* AM1, which was unable to grow on either methanol or ethanol, was utilized as the expression host strain (Vu et al., 2016). Plasmids were transferred in *Escherichia coli* Top10 and host strain through *E. coli* helper strain PRK2013 (Marx and Lidstrom, 2001; Huang et al., 2019). The three-way conjugation was carried out on LB plates as described before (Chistoserdova et al., 2007). Plasmid-borne function and counter-selection for *E. coli* presence were performed using the previous starvation method (Huang et al., 2019). *Methylorubrum extorquens* AM1 was selected on minimal medium plates supplemented with ethanol (0.15% v/v) as a substrate, while tetracycline (10 μg/mL) and rifampicin (50 μg/mL) were added.

### Protein expression and purification

For protein expression, 300 mL cultures were grown in shake flasks with succinate (0.2% w/v) to late exponential phase and collected by centrifugation at 5,000 *g* for 15 min. Pellets were transferred to 100 mL of fresh minimal medium supplemented with ethanol (0.15% v/v) and Ca^2+^ (1 mM), and they were incubated for 48 h at 30°C with shaking at 200 rpm. As mentioned above, cells were harvested by centrifugation at 5,000 *g* for 15 min at 4°C, either used immediately or stored at −80°C. The cell precipitation was resuspended in a start buffer (100 mM Tris–HCl, pH 9.0, 150 mM NaCl, 1 mM DTT, 0.2 mM PMSF) and was disrupted on ice with an ultrasonic crusher (SCIENIZ, Ningbo, China). The supernatant was collected by centrifugation at 11,000 *g* for 15 min at 4°C. The supernatants were mixed with 5 volumes of the start buffer and 1 volume of pre-balanced Ni-NTA agarose (Qiagen), and these mixtures were shaken for 15 min at 4°C to enhance the specificity of binding. Then the mixtures were loaded onto empty PD-10 columns (GE Healthcare). After two successive wash steps with 5 volumes of the start buffer and 3 volumes of the wash buffer (100 mM Tris–HCl pH 9.0, 150 mM NaCl, 30 mM imidazole, and 1 mM DTT), the elution step was carried out using the elution buffer (100 mM Tris–HCl pH 9.0, 150 mM NaCl, 250 mM imidazole, and 1 mM DTT). Protein samples were desalted and concentrated by a series of dilution/concentration steps, using 50 kDa Amicon Ultra centrifugal filter units (Millipore), until the concentration of imidazole reached below 1 μM. The protein was separated by 12.5% SDS-PAGE gel electrophoresis, and the concentration was measured by the method of Bradford with bovine serum albumin (BSA) as a standard (Zhai et al., 2022).

### Ethanol dehydrogenase assay

Ethanol dehydrogenase activity was measured by monitoring the phenazine methosulfate (PMS)-mediated reduction of 2,6-dichlorophenol-indophenol (DCPIP) (ε_600_ = 21.9 mM^−1^ cm^−1^) (Jahn et al., 2020). Initially, all assays were carried out at pH 9.0 following the classic assay (Anthony and Zatman, 1967), the standard reaction mixture containing: 100 mM Tris–HCl pH 9.0, 45 mM NH_4_Cl, 1 mM PMS, 150 μM DCPIP, 10 mM substrates, and 3–10 μL of pure protein preparation (0.5–3.0 mg/mL protein). Assays were performed at room temperature (approximately 26°C) in a total volume of 0.8 mL plastic cuvettes (1 cm path length). One unit (U) of specific enzyme activity was defined as 1 μmol DCPIP reduced per minute (determined at 600 nm) and was expressed as a unit per milligram of protein.

### Functional characterization

Substrate specificities were determined for several alcohols and aldehydes using optimal assay conditions, where substrates were supplied at 10 mM. Methanol, ethanol, and acetaldehyde were purchased from Traditional Chinese medicine, and other substrates were purchased from Macklin. To maintain optimum pH values, standard buffers were used as follows: 100 mM sodium/potassium phosphate (pH range 6.0–7.5), 100 mM Tris–HCl (pH range 7.5–9.0), 100 mM Gly-NaOH (pH range 9.0–10.0). The effect of ammonia was examined in the standard assay including or omitting 45 mM NH_4_Cl. The optimum temperature of the enzyme was determined with different temperatures varying from 25°C to 70°C.

The influence of metal ions on the enzyme activity was determined by adding 10 mM of cations (Na^+^, K^+^, Zn^2+^, Co^2+^, Cu^2+^, Ni^2+^, Ca^2+^, Mg^2+^, Sr^2+^, Ba^2+^, Mn^2+^, La^3+^, Nd^3+^, Sm^3+^, Gd^3+^, Yb^3+^) to the standard reaction buffer. The influences of organic solvents and detergents on enzyme activity were examined by using the presence of 10% (v/v) acetonitrile, 10% (v/v) Triton X-100, 10% (v/v) Triton X-114, 10% (v/v) Tween-20, 10% (v/v) Tween-80, and 10% (v/v) SDS. The enzyme activity assay was carried out under optimal standard reaction buffer with 100 mM Tris–HCl (pH 9.0) at 30°C, and the enzyme activity in the blank group was defined as 100% without additives.

### Enzyme kinetics

Kinetic parameters were determined using the respective optimal assay conditions using varying concentrations of selected substrates (0.001 to 1 mM). The values of *K*_m_ and *V*_max_ were obtained using the Michaelis–Menten equation, with GraphPad Software (GraphPad prism5, United States) (Zhai et al., 2022). Averages of kinetic constants were determined for each enzyme based on three biologically independent experiments.

### Structure prediction and visualization

The AlphaFlod was used to predict the protein structure (Jumper et al., 2021), and the full PedE_M.s. sequence was provided as an input. In addition, the predicted tertiary structure was finalized by comparing to the Ca^2+^-dependent EDH from *Pseudomonas aeruginosa* (PDB ID:1FLG). Furthermore, all the tertiary structures were visualized by PyMOL (Schrödinger, 2017). (Version 2.0 Schrödinger LLC).

## Results

### Cloning, expression, and purification of PedE_M.s.

**Figure 1:**
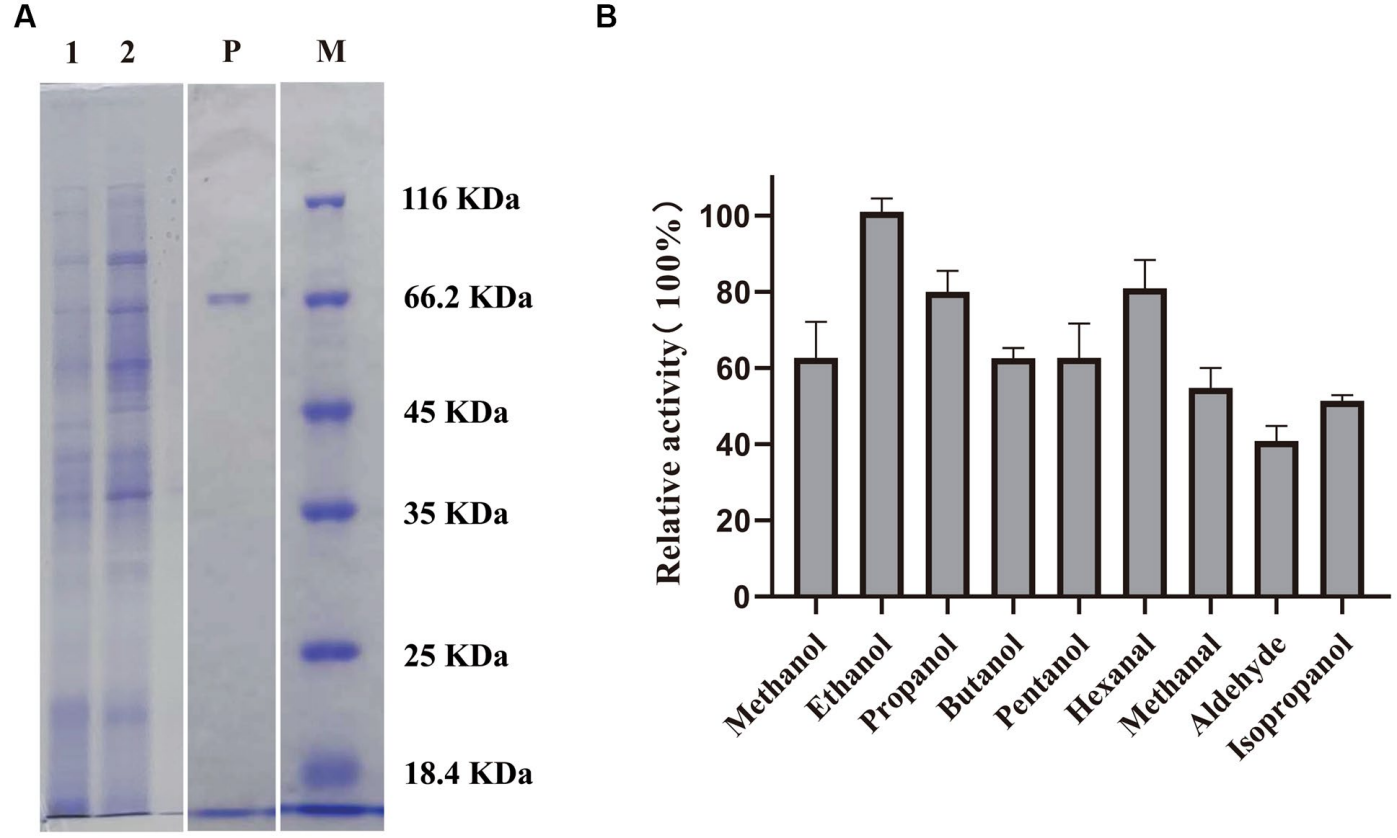
**(A)** SDS–PAGE analysis of PedE_M.s.. M: Marker; lane 1: cell lysate; lane 2: supernatant of the sonication product after centrifugation; lane P: purified PedE_M.s.. **(B)** Relative activity of PedE_M.s. to several alcohols and aldehydes. The most active substrate was defined as 100%. The relative activity of PedE_M.s. against various substrates was determined based on three biologically independent experiments. The error bars are shown in the figure.

Revealed by nucleotide sequence analysis, an open reading frame of 1896 bp in *Methylopila* sp. M107 encodes a protein of 631 amino acids with a theoretical molecular weight of 69.4 kDa. This protein had been named as PedE_M.s.. Based on NCBI and PDB databases, phylogenetic tree sequence analysis showed that PedE_M.s. belongs to the methanol/ethanol family PQQ-dependent dehydrogenase (Supplementary Figure S1). And PedE_M.s. was predicted to possess typical characteristics of ADHs, as observed in other members of the ExaA/PedE-type ADHs. To explore the physicochemical characterizations of this enzyme, the sequence of the PedE_M.s. was successfully constructed into the modified pCM80 vector with a C-terminal 8xHis tag, and the recombinant proteins were expressed in *Methylorubrum extorquens* AM1 cells. After the cells were grown, the expressed PedE_M.s. proteins were extracted and purified by His-tag affinity purification to 95% homogeneity. The purified proteins were buffer-exchanged to make the imidazole concentration below 1 μM and further concentrated using the 50 kDa Amicon Ultra centrifugal filter unit. Concentrated PedE_M.s. (1 ± 0.5 mg) was obtained from 300 mL cultures and its molecular weight was shown by SDS-PAGE (Figure 1A).

### Biochemical characterization of PedE_M.s.

**Figure 2:**
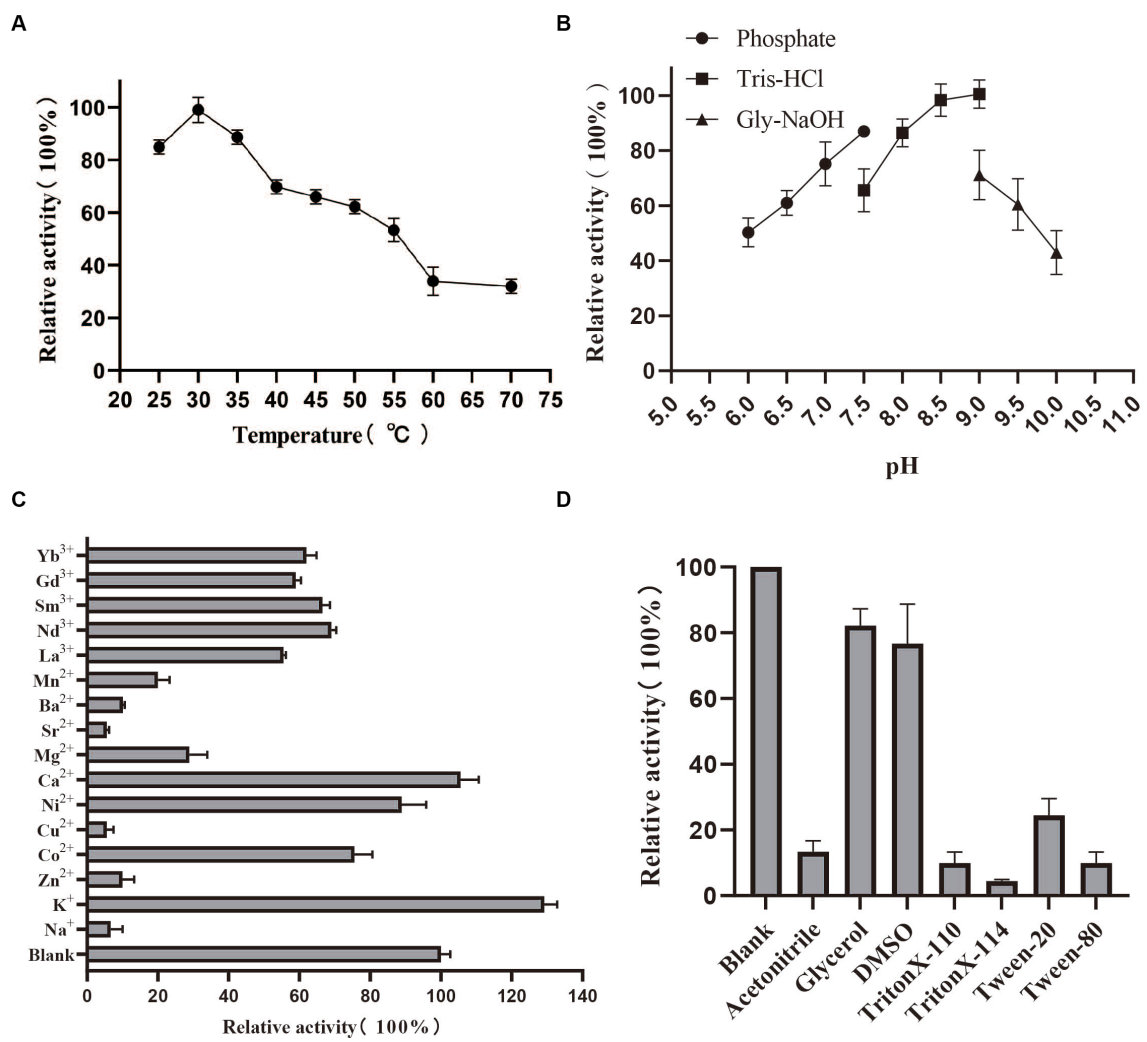
Biochemical characterization of PedE_M.s.. The effect of temperature **(A)** and pH **(B)** on enzyme activity of PedE_M.s.. All values were measured under the optimal substrate. The value obtained at 30°C and pH 9.0 was taken as 100%, respectively. Effects of different metal ions (10 mM) **(C)**, detergents 10% (v/v), and organic solvents 10% (v/v) **(D)** on the enzymatic activity. All values were measured using optimal substrate, temperature and pH. Blank group indicated that no additional metal ions, detergents, and organic solvents are added. The values of blank were taken as 100%. All values in this figure were measured through three biologically independent experiments, and the error bars are shown in the figure.

By measuring the activity of PedE_M.s. on several alcohols and aldehydes, we found that PedE_M.s. displayed the highest activity on ethanol (Figure 1B; Supplementary Table S1), which demonstrated that PedE_M.s. was a kind of EDH. The activity of PedE_M.s. was maintained 45–90% in a standard sodium/potassium phosphate buffer (pH range 6.0–7.5), 65–100% in a Tris–HCl buffer (pH range 7.5–9.0), and 45–70% in a Gly-NaOH buffer (pH range 9.0–10.0) (Figure 2B). Moreover, PedE_M.s. could maintain more than 80% catalytic activity toward ethanol at a temperature range of 25°C–35°C and a pH range from pH 7.5 to pH 9.0 (Figures 2A,B). And it was noteworthy that PedE_M.s. showed the highest catalytic activity at 30°C and pH 9.0 (Figures 2A,B).

### Effect of cations, organic solvents, and detergents on PedE_M.s. activity

In order to understand the potential applications of PedE_M.s., we investigated the relative activity of PedE_M.s. after adding different cations, detergents, and organic solvents to the reaction buffer at the optimum temperature and pH (Figures 2C,D). The activity of PedE_M.s. was only about 10–30% retained with Na^+^, Zn^2+^, Cu^2+^, Mg^2+^, Sr^2+^, Ba^2+^, Mn^2+^, and 50–80% activity remained with Co^2+^, Ni^2+^, La^3+^, Nd^3+^, Sm^3+^, Gd^3+^, Yb^3+^, and exceeded 100% with K^+^, Ca^2+^ (Figure 2C). Moreover, the activity of PedE_M.s. was compared with the blank under the addition of 10% (v/v) organic solvents (acetonitrile, glycerol, DMSO) or 10% (v/v) detergents (Triton X-100, Triton X-T114, Tween-20, and Tween-80), its activity was attenuated. Unexpectedly, the activity decreased by 80–95% after the addition of acetonitrile, Tween-80, Triton X-100 and Triton X-114 (Figure 2D). In addition, glycerol and DMSO have a less inhibitory effect on PedE_M.s. because the PedE_M.s. activity remained at 80% after adding glycerol and DMSO. Enzyme activity was found to be reduced due to organic solvents and detergents, which indicated that these reagents might have affected the conformation of PedE_M.s.. Therefore, these reagents were found unsuitable for crystallization of the enzyme.

### Enzyme kinetics

The kinetic parameters for various substrates with PedE_M.s. were investigated (Table 1; Supplementary Figure S2). Similar to most ADHs, PedE_M.s. could oxidize many alcohols and aldehydes. Ethanol was the preferred substrate for this enzyme. PedE_M.s. also showed a relatively higher catalytic rate on methanol and propanol, with *K*_cat_/*K*_m_ values of 22.21 ± 8.23 and 23.13 ± 10.96 for methanol and propanol respectively, compared with 59.49 ± 20.43 for ethanol. The *K*_cat_/*K*_m_ value of PedE_M.s. for ethanol was 2 to 8 times higher than other alcohols and aldehydes (Table 1), suggesting that PedE_M.s. has the highest catalytic efficiency for ethanol. Specifically, PedE_M.s. also had a higher affinity on ethanol than other PedE-type enzymes (Supplementary Table S2).

**Table 1:** Summary of the kinetic parameters of distinct substrates used in this study.

Source	*Methylopila* sp. M107		
Catalytic properties	*V*_max_ (U/mg)	*K*_m_ (mM)	*K*_eff_^#^ (s^−1^ mM^−1^)
Methanol	0.24 ± 0.01	0.025 ± 0.006	22.21 ± 8.23
Ethanol	0.72 ± 0.04	0.028 ± 0.006	59.49 ± 20.43
Propanol	0.25 ± 0.03	0.025 ± 0.006	23.13 ± 10.96
Butanol	0.29 ± 0.02	0.039 ± 0.015	17.20 ± 12.68
Pentanol	0.22 ± 0.02	0.049 ± 0.014	10.39 ± 5.47
Hexanol	0.26 ± 0.02	0.079 ± 0.014	7.61 ± 2.36
Formaldehyde	0.24 ± 0.03	0.039 ± 0.021	14.24 ± 20.46
Aldehyde	0.13 ± 0.01	0.041 ± 0.027	7.33 ± 15.80
Isopropanol	0.24 ± 0.03	0.035 ± 0.012	15.86 ± 11.30

Averages of kinetic constants were determined for each enzyme based on three biologically independent experiments.

### Active sites and structure prediction

**Figure 3:**
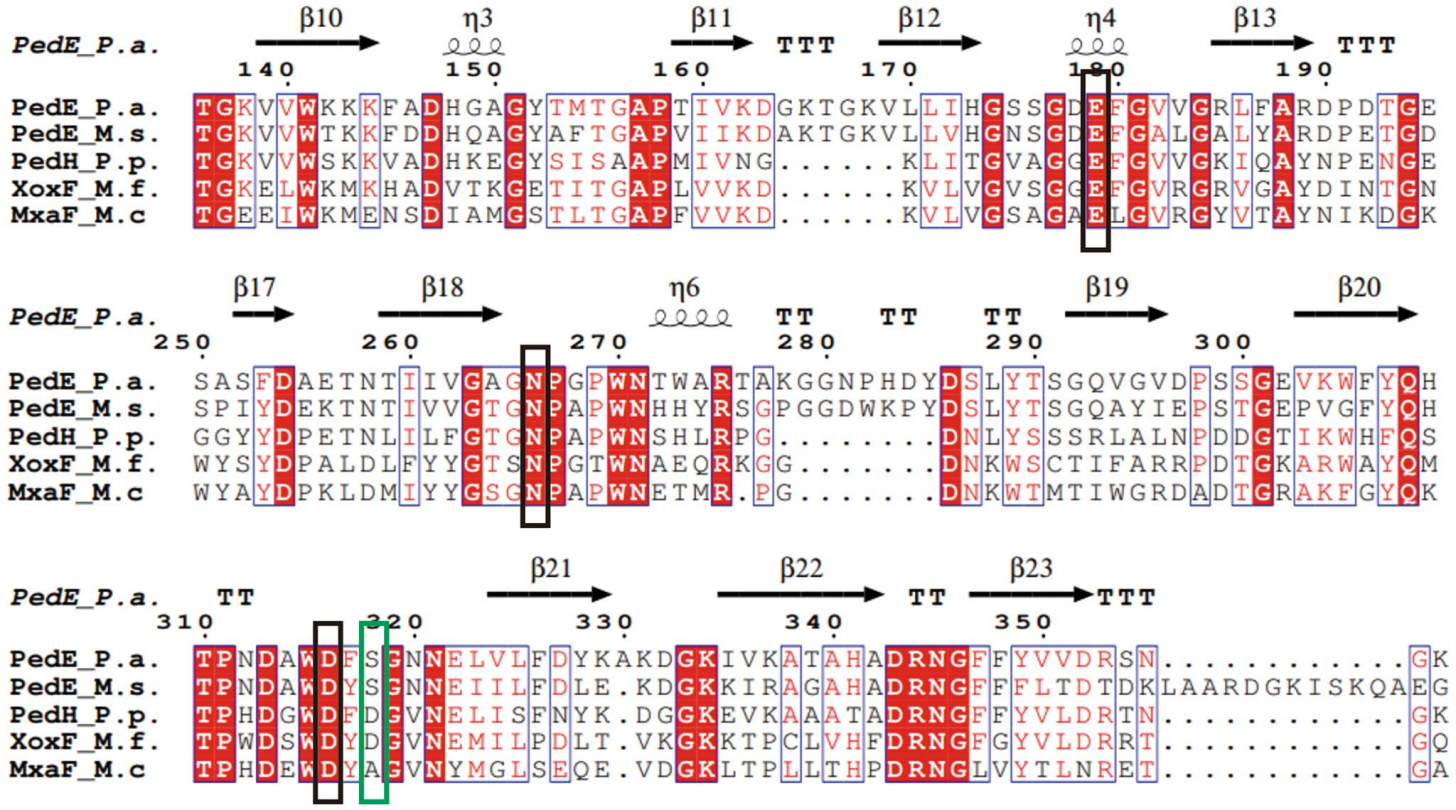
Multiple sequence alignment of PedE_M.s. and other members of the methanol/ethanol family PQQ-dependent dehydrogenase. The sequences included Ca^2+^-dependent EDH PedE_P.a. from *Pseudomonas aeruginosa* (PDB ID:1FLG), Ca^2+^-dependent EDH PedE_M.s. from *Methylopila* sp. M107, La^3+^-dependent EDH PedH_P.p. from *Pseudomonas putida* KT2440 (PDB ID:6ZCW), Ce^3+^-dependent MDH XoxF_M.f. from *Methylacidiphilum fumariolicum* Solv (PDB ID:4MAE), and Ca^2+^-dependent MDH MxaF_M.c. from *Methylococcus capsulatus* (PDB ID:4TQO). The conserved active site in the methanol/ethanol family PQQ-dependent dehydrogenase were marked with a black box. The differences between distinct types of ADH were marked with a green box. Secondary structure elements of PedE_P.a. is displayed at the top (helices with squiggles, β-strands with arrows and turns with TT letters). Red boxes indicate identical residues. Blue boxes show that residues are similar and relatively conservative. White characters indicate the same residues, red characters represent similar residues, and black characters point to lower consistency of the residues.

**Figure 4:**
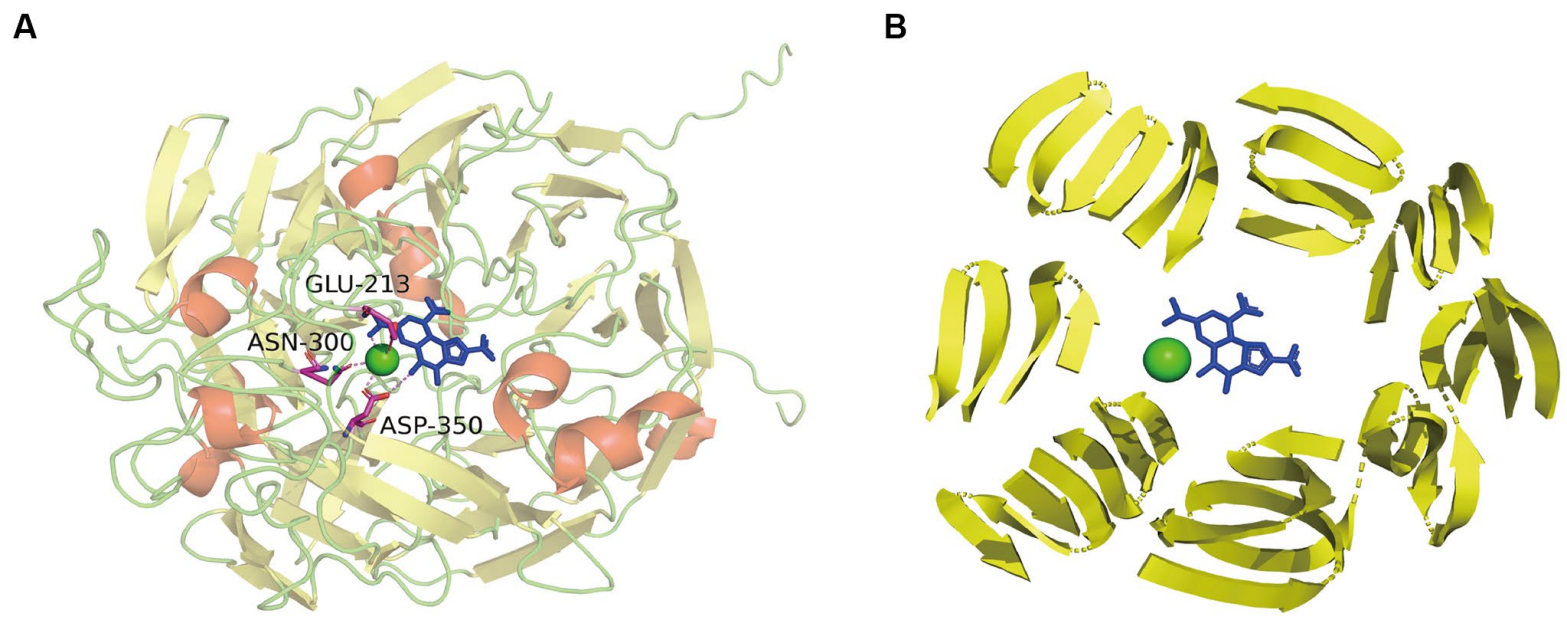
**(A)** The overall structure of PedE_M.s. and interplay between active sites and Ca^2+^-PQQ. α-Helices were shown in red; β-strands were denoted as yellow arrows; cyan dots represented calcium; the blue residue indicated the PQQ group. Residues of active sites were shown in sticks: magenta, C; blue, N; red, O. The interaction between Ca^2+^-PQQ and conserved amino acids were shown by violet dotted line. **(B)** Presentation of 8-bladed propeller-like super-barrel structure (yellow arrows) and the Ca^2+^-PQQ active center.

Sequence alignment of PedE_M.s. with the methanol/ethanol family PQQ-dependent dehydrogenase showed that PedE_M.s. exhibited the highest sequence identity of 67.91% with Ca^2+^-dependent EDH PedE_P.a. from *Pseudomonas aeruginosa* (PDB ID:1FLG). Sequence alignments (Larkin et al., 2007; Robert and Gouet, 2014) further displayed that PedE_M.s. contained three typical catalytic residues, which were Glu213, Asn300, Asp350 (Figure 3). Compared to La^3+^-dependent ADHs, the 352th amino acid of PedE_M.s. was Ser352 instead of Asp352, based on presence of different metal ions in the active center (Keitel et al., 2000). Protein structure was predicted by entering the sequence of PedE_M.s. and the reference structure of PedE_P.a. (PDB ID:1FLG) into AlphaFlod. It is evident from the results that PedE_M.s. constitutes an 8-bladed propeller-like super-barrel (Keitel et al., 2000) and a Ca^2+^-PQQ active center containing three catalytic residues Glu213, Asn300, and Asp350 (Figure 4).

## Discussion

ADHs are widely distributed in microorganisms, plants, humans, and animals. The current focus is mainly on studying NAD^+^-dependent ADHs in humans and animals, while only a few studies have focused on PQQ-dependent ADHs in microorganisms. The majority of studies on PQQ-dependent ADHs have centered on microorganisms such as acetic acid bacteria, *Pseudomonas putida* and metamorphic bacteria (Yakushi and Matsushita, 2010; Trček and Matsushita, 2013; Aquino Neto et al., 2015; Wehrmann et al., 2017; Guo et al., 2019), the primary focus of these studies is to explore the relevance of PQQ-dependent ADH characteristics in production applications. However, gram-negative methylotrophs growing by using monomethyl compounds as carbon sources typically possess a PQQ-dependent alcohol dehydrogenase (ADH). These bacteria play a significant role in the global carbon and nitrogen cycles due to their unique PQQ-dependent ADHs. Our study found that PedE_M.s. derived from methylotroph *Methylopila* sp. M107, is a novel member of the methanol/ethanol family PQQ-dependent dehydrogenase. It exhibits broad substrate specificity, with ethanol being the optimal substrate, while the *K*_m_ value is 0.028 mM. Notably, compared to PedE in *P.putida* KT2440, *Gluconobacter* sp. 33, *Geobacillus stearothermophilus* DSM 2334, and *Frateuria aurantia* LMG 1558 T, the affinity of PedE_M.s. for ethanol increased by at least 3 times under optimal conditions without the addition of other reagents (Trček and Matsushita, 2013; Aquino Neto et al., 2015; Wehrmann et al., 2017; Guo et al., 2019; Supplementary Table S2), which is valuable to the global carbon and nitrogen cycles and industrial production of fuel cell (Zulic and Minteer, 2011). Biofuel cells can be more cost effective, environmentally friendly, and flexible systems than conventional fuel cells. Biofuel cells use biocatalysts (organisms, organelles, or enzymes) to convert biochemical energy into electrical energy (Cooney et al., 2008). Therefore, the enrichment of methanol/ethanol family PQQ-dependent dehydrogenase members is crucial to solve the problems such as global resource shortage and environmental pollution. Besides, the optimal temperature and pH of PedE_M.s. were 30°C and pH 9.0, respectively. However, even at the same pHs, the activities were different in different buffers. Our explanation for this is that different buffers contain different ions that may affect the configuration of the enzyme or the degree of substrate dissociation resulting in varying activities (Ahmad et al., 2023).

The enzymatic properties of PedE_M.s. are similar to other PedE-type ADHs. However, its outstanding characteristic is that PedE_M.s. retains 60–80% activity even in the presence of 10 mM lanthanides, on the contrary, other PedE-type ADHs are inactive in the presence of lanthanides (Wehrmann et al., 2017). The presence of negatively charged residues (Asp/Glu) in the metal ion binding cavity of La^3+^-dependent ADHs and Ca^2+^-dependent ADHs had been shown in previous studies (Prejanò et al., 2017). To complete the coordination environment, La^3+^-dependent ADHs utilized additional aspartic acid residues, which enhanced the limited flexibility in the typical binding cavity of Ca^2+^ (Prejanò et al., 2017). However, PedE_M.s. did not have any metal ion binding sites that differ from other PedE-type ADHs, and no aspartic acid residue located in rare earth coordination was found on comparing the sequence and structure of PedE_M.s. with other ADHs. Why does PedE_M.s. functions even in the presence of 10 mM lanthanides? It is worth noting that even under the condition of an XoxF-type promoter, MxaF-MDH can still be translated and expressed (Wehrmann et al., 2017), which demonstrated that the expression of Ca^2+^-dependent ADHs can also be induced by XoxF-type promoter. Moreover, studies have shown the existence of an additional La^3+^-responsive regulatory module (Chu et al., 2016), and this La^3+^-responsive regulatory module might be activated by XoxF-type promoter to change the conformation of PedE_M.s.. Therefore, we assume an association between La^3+^-responsive regulatory module and the XoxF-type promoter used in vector construction, but there is no conclusive evidence for this conjecture which is one of the limitations of our experiment, and we will change promoter types to express and purify PedE_M.s. in future studies. As well, it had been shown that the binding modes of PQQ with metal ions were only XoxF-type and MxaF-type (Vu et al., 2016). However, their binding modes will have a key effect on the activity of ADHs. Studies have shown that La^3+^ binds to PQQ in the chelate binding mode in XoxF-type while becoming more loosely associated with PQQ in MxaF-type (Vu et al., 2016). Therefore, the binding modes of PQQ with La^3+^ in PedE_M.s. affect the enzyme activity. The unique binding modes between PQQ and La^3+^ in PedE_M.s. might increase the enzyme’s activity in the presence of lanthanides. However, these particular binding modes are still needed further studies.

Another significant inference of this study is that there was a metabolic interdependence between La^3+^-dependent ExaF-type and Ca^2+^-dependent PedE-type EDHs, and that PedE-type might be converted to ExaF-type EDHs under stressful environmental incubation (Supplementary Table S1). Previous research reported that La^3+^-dependent PQQ-ADHs were considered ancestral and more widespread than their Ca^2+^-dependent homologs (Keltjens et al., 2014; Vekeman et al., 2016). This suggested that Ca^2+^-dependent enzymes may have evolved to colonize different environmental niches in which lanthanide availability is less effective. Therefore, when lanthanide concentration increases in environment, PedE_M.s. may transform PedE-type EDH into ExaF-type EDH through some mechanisms. Metabolic interdependence is considered a driving force of species’ coexistence and interrelationships in different microbial communities, affecting their structure and functions (Estrela et al., 2013; Zelezniak et al., 2015), which is of particular relevance in the context of studies of Ca^2+^-dependent ADHs active on lanthanides. Organic alcohols and related oxidation products are not only crucial intermediates in the global carbon cycle, but they can also exhibit additional functions, including signaling and growth inhibition (Bitas et al., 2013; Garbeva et al., 2015). A recent study mentioned that methanotrophic bacteria shifted their gene expression from XoxF-type to MxaF-type MDHs under co-culture conditions for methanogenic and non-methanogenic bacteria in the presence of methane and lanthanides (Krause et al., 2017). Although the underlying mechanism of shifting phenomenon is still unclear, based on our results, we can speculate that similar interactions are not limited to methanotrophs but rather have associations within a broader ecological context.

ADHs catalyzed alcoholic oxidation, remaining consistent with the reduction of PQQ by methanol and the release of formaldehyde. This is followed by two successive single-electron transfers to cytochrome *c*_L_, during which PQQH_2_ is oxidized back to quinone via the radical semiquinone (Frank et al., 1988). Based on the above conclusion, we assume that the first step of the reaction is proton extraction from the alcohol by the active site Glu213, Asn300, Asp350. According to the mechanism described by Afolabi et al (Afolabi et al., 2001), Ca^2+^ acts as Lewis acids by coordinating with the C5 carbonyl oxygen of PQQ, thereby stabilizing the electrophilic C5 and allowing it to be attacked by oxygen ions or hydrides. The presence of Ca^2+^ plays an essential role in increasing the nucleophilicity of the C5 atom of PQQ. However, the activities of PedE_M.s. were improved by K^+^ and Ca^2+^. This might stabilize the spatial structure and charge of the transition state by directly coordinating with its functional groups (Gohara and Di Cera, 2016). Alternatively, they might bind to sites which are not in direct contact with the substrate and improve the catalytic activity through conformational transition (Afolabi et al., 2001).

This study screened PedE_M.s. from *Methylopila* sp. M107, which exhibited a broad range of substrate specificity, with ethanol being the optimum substrate. Concomitantly, structure prediction revealed a typical 8-bladed propeller-like super-barrel structure in the methanol/ethanol family PQQ-dependent dehydrogenase. This study has the potential to significantly improve our understanding of the molecular mechanisms of methanol/ethanol family PQQ-dependent dehydrogenase. Additionally, this understanding of the optimal physiological and biochemical basis of this enzyme could contribute to improve the industrial application value of PedE_M.s.. More importantly, the application of ADHs in biosensors, environmental protection, and other industries are expanded.

## Data availability statement

The original contributions presented in the study are included in the article/Supplementary material, further inquiries can be directed to the corresponding author.

## Author contributions

JH and YX conceived the study and designed the experiments. YX, KW, XZ, and HC carried out the experiments, analyzed the data, and prepared the figures. YX wrote the draft. YX, JH, SB, QW, and ZY participation in discussion and edited the final manuscript. All authors contributed to this manuscript and approved the final submitted version.

## Funding

The work was supported by the National Natural Science Foundation of China (32000054 and 32170071) and the Natural Science Foundation of Hunan Province (2023JJ30651).

## Conflict of interest

The authors declare that the research was conducted in the absence of any commercial or financial relationships that could be construed as a potential conflict of interest.

## Publisher’s note

All claims expressed in this article are solely those of the authors and do not necessarily represent those of their affiliated organizations, or those of the publisher, the editors and the reviewers. Any product that may be evaluated in this article, or claim that may be made by its manufacturer, is not guaranteed or endorsed by the publisher.

## Footnotes

^1^ https://blast.ncbi.nlm.nih.gov/Blast.cgi

^2^ http://www.clustal.org/

^3^ https://espript.ibcp.fr/ESPript/ESPript/esp_tutorial.php

## References

[ref1] Afolabi P. R. Mohammed F. Amaratunga K. Majekodunmi O. Dales S. L. Gill R.. (2001). Site-directed mutagenesis and X-ray crystallography of the PQQ-containing quinoprotein methanol dehydrogenase and its electron acceptor, cytochrome c(L). Biochemistry 40, 9799–9809. doi: 10.1021/bi002932l, PMID: 11502173

[ref2] Ahmad R. Rizaldo S. Gohari M. Shanahan J. Shaner S. E. E. Stone K. L. L.. (2023). Buffer effects in zirconium-based UiO metal-organic frameworks (MOFs) that influence enzyme immobilization and catalytic activity in enzyme/MOF biocatalysts. ACS Omega 8, 22545–22555. doi: 10.1021/acsomega.3c00703, PMID: 37396281 PMC10308582

[ref3] Anderson D. J. Lidstrom M. E. (1988). The moxFG region encodes four polypeptides in the methanol-oxidizing bacterium Methylobacterium sp. strain AM1. J. Bacteriol. 170, 2254–2262. doi: 10.1128/jb.170.5.2254-2262.1988, PMID: 3129405 PMC211115

[ref4] Anthony C. (2001). Pyrroloquinoline quinone (PQQ) and quinoprotein enzymes. Antioxid. Redox Signal. 3, 757–774. doi: 10.1089/15230860152664966 11761326

[ref5] Anthony C. Williams P. (2003). The structure and mechanism of methanol dehydrogenase. Biochimica et Biophysica Acta 1647, 18–23. doi: 10.1016/S1570-9639(03)00042-6 12686102

[ref6] Anthony C. Zatman L. J. (1967). The microbial oxidation of methanol. Purification and properties of the alcohol dehydrogenase of Pseudomonas sp. M27. Biochem. J. 104, 953–959. doi: 10.1042/bj1040953, PMID: 6058112 PMC1271237

[ref7] Aquino Neto S. Hickey D. P. Milton R. D. De Andrade A. R. Minteer S. D. (2015). High current density PQQ-dependent alcohol and aldehyde dehydrogenase bioanodes. Biosens. Bioelectron. 72, 247–254. doi: 10.1016/j.bios.2015.05.011, PMID: 25988787

[ref8] Aquino Neto S. Milton R. D. Hickey D. P. De Andrade A. R. Minteer S. D. (2016). Membraneless enzymatic ethanol/O-2 fuel cell: transitioning from an air-breathing Pt-based cathode to a bilirubin oxidase-based biocathode. J. Power Sources 324, 208–214. doi: 10.1016/j.jpowsour.2016.05.073

[ref9] Bitas V. Kim H.-S. Bennett J. W. Kang S. (2013). Sniffing on microbes: diverse roles of microbial volatile organic compounds in plant health. Mol. Plant-Microbe Interact. 26, 835–843. doi: 10.1094/MPMI-10-12-0249-CR, PMID: 23581824

[ref10] Chistoserdova L. (2015). Methylotrophs in natural habitats: current insights through metagenomics. Appl. Microbiol. Biotechnol. 99, 5763–5779. doi: 10.1007/s00253-015-6713-z, PMID: 26051673

[ref11] Chistoserdova L. Crowther G. J. Vorholt J. A. Skovran E. Portais J.-C. Lidstrom M. E. (2007). Identification of a fourth formate dehydrogenase in *Methylobacterium extorquens* AM1 and confirmation of the essential role of formate oxidation in methylotrophy. J. Bacteriol. 189, 9076–9081. doi: 10.1128/JB.01229-07, PMID: 17921299 PMC2168636

[ref12] Chistoserdova L. Kalyuzhnaya M. G. (2018). Current trends in Methylotrophy. Trends Microbiol. 26, 703–714. doi: 10.1016/j.tim.2018.01.011, PMID: 29471983

[ref13] Chistoserdova L. Kalyuzhnaya M. G. Lidstrom M. E. (2009). The expanding world of methylotrophic metabolism. Annu. Rev. Microbiol. 63, 477–499. doi: 10.1146/annurev.micro.091208.073600, PMID: 19514844 PMC2827926

[ref14] Choi O. Kim J. Kim J.-G. Jeong Y. Moon J. S. Park C. S.. (2008). Pyrroloquinoline quinone is a plant growth promotion factor produced by *Pseudomonas fluorescens* B16. Plant Physiol. 146, 657–668. doi: 10.1104/pp.107.112748, PMID: 18055583 PMC2245851

[ref15] Chu F. Beck D. A. C. Lidstrom M. E. (2016). MxaY regulates the lanthanide-mediated methanol dehydrogenase switch in *Methylomicrobium buryatense*. PeerJ 4:e2435. doi: 10.7717/peerj.2435, PMID: 27651996 PMC5018670

[ref16] Cooney M. J. Svoboda V. Lau C. Martin G. Minteer S. D. (2008). Enzyme catalysed biofuel cells. Energy Environ. Sci. 1, 320–337. doi: 10.1039/b809009b

[ref17] Estrela S. Brown S. P. Allesina S. (2013). Metabolic and demographic feedbacks shape the emergent spatial structure and function of microbial communities. PLoS Comput. Biol. 9:e1003398. doi: 10.1371/journal.pcbi.1003398, PMID: 24385891 PMC3873226

[ref18] Frank J. Dijkstra M. Duine J. A. Balny C. (1988). Kinetic and spectral studies on the redox forms of methanol dehydrogenase from Hyphomicrobium X. Eur. J. Biochem. 174, 331–338. doi: 10.1111/j.1432-1033.1988.tb14102.x, PMID: 3289922

[ref19] Garbeva P. Schmidt R. Cordovez V.. (2015). Volatile affairs in microbial interactions. ISME J. 9, 2329–2335. doi: 10.1038/ismej.2015.42 26023873 PMC4611499

[ref20] Gohara D. W. Di Cera E. (2016). Molecular mechanisms of enzyme activation by monovalent cations. J. Biol. Chem. 291, 20840–20848. doi: 10.1074/jbc.R116.737833, PMID: 27462078 PMC5076497

[ref22] Guo X. Feng Y. Wang X. Liu Y. Liu W. Li Q.. (2019). Characterization of the substrate scope of an alcohol dehydrogenase commonly used as methanol dehydrogenase. Bioorg. Med. Chem. Lett. 29, 1446–1449. doi: 10.1016/j.bmcl.2019.04.025, PMID: 31006524

[ref23] Gvozdev A. R. Tukhvatullin I. A. Gvozdev R. I. (2012). Quinone-dependent alcohol dehydrogenases and FAD-dependent alcohol oxidases. Biochemistry (Mosc) 77, 843–856. doi: 10.1134/S0006297912080056, PMID: 22860906

[ref24] Huang J. Yu Z. Groom J. Cheng J.-F. Tarver A. Yoshikuni Y.. (2019). Rare earth element alcohol dehydrogenases widely occur among globally distributed, numerically abundant and environmentally important microbes. ISME J. 13, 2005–2017. doi: 10.1038/s41396-019-0414-z, PMID: 30952993 PMC6775964

[ref25] Ivnitski D. Atanassov P. Apblett C. (2007). Direct Bioelectrocatalysis of PQQ-dependent glucose dehydrogenase. Electroanalysis 19, 1562–1568. doi: 10.1002/elan.200703899

[ref26] Jahn B. Jonasson N. S. W. Hu H. Singer H. Pol A. Good N. M.. (2020). Understanding the chemistry of the artificial electron acceptors PES, PMS, DCPIP and Wurster’s blue in methanol dehydrogenase assays. J. Biol. Inorg. Chem. 25, 199–212. doi: 10.1007/s00775-020-01752-9, PMID: 32060650 PMC7082304

[ref27] Jumper J. Evans R. Pritzel A. Green T. Figurnov M. Ronneberger O.. (2021). Highly accurate protein structure prediction with AlphaFold. Nature 596, 583–589. doi: 10.1038/s41586-021-03819-2, PMID: 34265844 PMC8371605

[ref28] Keitel T. Diehl A. Knaute T. Stezowski J. J. Höhne W. Görisch H. (2000). X-ray structure of the quinoprotein ethanol dehydrogenase from *Pseudomonas aeruginosa*: basis of substrate specificity. J. Mol. Biol. 297, 961–974. doi: 10.1006/jmbi.2000.3603, PMID: 10736230

[ref29] Keltjens J. T. Pol A. Reimann J. Op den Camp H. J. M. (2014). PQQ-dependent methanol dehydrogenases: rare-earth elements make a difference. Appl. Microbiol. Biotechnol. 98, 6163–6183. doi: 10.1007/s00253-014-5766-8, PMID: 24816778

[ref30] Killgore J. Smidt C. Duich L. Romero-Chapman N. Tinker D. Reiser K.. (1989). Nutritional importance of pyrroloquinoline quinone. Science 245, 850–852. doi: 10.1126/science.2549636, PMID: 2549636

[ref31] Krause S. M. B. Johnson T. Samadhi Karunaratne Y. Fu Y. Beck D. A. C. Chistoserdova L.. (2017). Lanthanide-dependent cross-feeding of methane-derived carbon is linked by microbial community interactions. Proc. Natl. Acad. Sci. U. S. A. 114, 358–363. doi: 10.1073/pnas.1619871114, PMID: 28028242 PMC5240692

[ref32] Larkin M. A. Blackshields G. Brown N. P. Chenna R. McGettigan P. A. McWilliam H.. (2007). Clustal W and Clustal X version 2.0. Bioinformatics 23, 2947–2948. doi: 10.1093/bioinformatics/btm404, PMID: 17846036

[ref33] Marx C. J. Lidstrom M. E. (2001). Development of improved versatile broad-host-range vectors for use in methylotrophs and other gram-negative bacteria. Microbiology (Reading) 147, 2065–2075. doi: 10.1099/00221287-147-8-2065 11495985

[ref34] Matsumura H. Umezawa K. Takeda K. Sugimoto N. Ishida T. Samejima M.. (2014). Discovery of a eukaryotic pyrroloquinoline quinone-dependent oxidoreductase belonging to a new auxiliary activity family in the database of carbohydrate-active enzymes. PLoS One 9:e104851. doi: 10.1371/journal.pone.0104851, PMID: 25121592 PMC4133262

[ref35] Morris C. J. Kim Y. M. Perkins K. E. Lidstrom M. E. (1995). Identification and nucleotide sequences of mxaA, mxaC, mxaK, mxaL, and mxaD genes from *Methylobacterium extorquens* AM1. J. Bacteriol. 177, 6825–6831. doi: 10.1128/jb.177.23.6825-6831.1995, PMID: 7592474 PMC177549

[ref36] Oberortner E. Cheng J.-F. Hillson N. J. Deutsch S. (2017). Streamlining the design-to-build transition with build-optimization software tools. ACS Synth. Biol. 6, 485–496. doi: 10.1021/acssynbio.6b00200, PMID: 28004921

[ref37] Oppermann U. C. Maser E. (2000). Molecular and structural aspects of xenobiotic carbonyl metabolizing enzymes. Role of reductases and dehydrogenases in xenobiotic phase I reactions. Toxicology 144, 71–81. doi: 10.1016/s0300-483x(99)00192-4, PMID: 10781873

[ref38] Pfeifenschneider J. Brautaset T. Wendisch V. F. (2017). Methanol as carbon substrate in the bio-economy: metabolic engineering of aerobic methylotrophic bacteria for production of value-added chemicals. Biofuels Bioprod. Biorefin. 11, 719–731. doi: 10.1002/bbb.1773

[ref39] Prejanò M. Marin T. Russo N. (2017). How can methanol dehydrogenase from Methylacidiphilum fumariolicum work with the alien CeIII ion in the active center? A Theoretical Study. Chem. Europ. J. 23, 8652–8657. doi: 10.1002/chem.201700381 28488399

[ref40] Puri A. W. Liu D. Schaefer A. L. Yu Z. Pesesky M. W. Greenberg E. P.. (2019). Interspecies chemical signaling in a methane-oxidizing bacterial community. Appl. Environ. Microbiol. 85, e02702–e02718. doi: 10.1128/AEM.02702-18, PMID: 30709826 PMC6585505

[ref41] Ramanavicius A. Ramanaviciene A. (2009). Hemoproteins in design of biofuel cells. Fuel Cells 9, 25–36. doi: 10.1002/fuce.200800052

[ref42] Razumiene J. Vilkanauskyte A. Gureviciene V. Barkauskas J. Meskys R. Laurinavicius V. (2006). Direct electron transfer between PQQ dependent glucose dehydrogenases and carbon electrodes: an approach for electrochemical biosensors. Electrochim. Acta 51, 5150–5156. doi: 10.1016/j.electacta.2006.03.058

[ref43] Robert X. Gouet P. (2014). Deciphering key features in protein structures with the new ENDscript server. Nucleic Acids Res. 42, W320–W324. doi: 10.1093/nar/gku316, PMID: 24753421 PMC4086106

[ref44] Sakuraba H. Yokono K. Yoneda K. Watanabe A. Asada Y. Satomura T.. (2010). Catalytic properties and crystal structure of quinoprotein aldose sugar dehydrogenase from hyperthermophilic archaeon *Pyrobaculum aerophilum*. Arch. Biochem. Biophys. 502, 81–88. doi: 10.1016/j.abb.2010.08.002 20692227

[ref45] Sarmiento-Pavía P. D. Sosa-Torres M. E. (2021). Bioinorganic insights of the PQQ-dependent alcohol dehydrogenases. J. Biol. Inorg. Chem. 26, 177–203. doi: 10.1007/s00775-021-01852-0, PMID: 33606117

[ref21] Schrödinger, L. L. C. (2017). The PyMOL Molecular Graphics System, Version 2.0. Available at: http://www.pymol.org/pymol

[ref46] Shirai T. Hung V. S. Morinaka K. Kobayashi T. Ito S. (2008). Crystal structure of GH13 alpha-glucosidase GSJ from one of the deepest sea bacteria. Proteins 73, 126–133. doi: 10.1002/prot.22044, PMID: 18398906

[ref47] Skovran E. Martinez-Gomez N. C. (2015). Microbiology. Just add lanthanides. Science 348, 862–863. doi: 10.1126/science.aaa9091 25999492

[ref48] Takeda K. Matsumura H. Ishida T. Samejima M. Ohno H. Yoshida M.. (2015). Characterization of a novel PQQ-dependent quinohemoprotein pyranose dehydrogenase from Coprinopsis cinerea classified into auxiliary activities family 12 in carbohydrate-active enzymes. PLoS One 10:e0115722. doi: 10.1371/journal.pone.0115722 25679509 PMC4332668

[ref49] Toyama H. Chistoserdova L. Lidstrom M. E. (1997). Sequence analysis of pqq genes required for biosynthesis of pyrroloquinoline quinone in *Methylobacterium extorquens* AM1 and the purification of a biosynthetic intermediate. Microbiology (Reading) 143, 595–602. doi: 10.1099/00221287-143-2-595, PMID: 9043136

[ref50] Toyama H. Mathews F. S. Adachi O. Matsushita K. (2004). Quinohemoprotein alcohol dehydrogenases: structure, function, and physiology. Arch. Biochem. Biophys. 428, 10–21. doi: 10.1016/j.abb.2004.03.037, PMID: 15234265

[ref51] Trček J. Jernejc K. Matsushita K. (2007). The highly tolerant acetic acid bacterium *Gluconacetobacter europaeus* adapts to the presence of acetic acid by changes in lipid composition, morphological properties and PQQ-dependent ADH expression. Extremophiles 11, 627–635. doi: 10.1007/s00792-007-0077-y, PMID: 17487444

[ref52] Trček J. Matsushita K. (2013). A unique enzyme of acetic acid bacteria, PQQ-dependent alcohol dehydrogenase, is also present in *Frateuria aurantia*. Appl. Microbiol. Biotechnol. 97, 7369–7376. doi: 10.1007/s00253-013-5007-6, PMID: 23760531

[ref53] Trcek J. Toyama H. Czuba J. Misiewicz A. Matsushita K. (2006). Correlation between acetic acid resistance and characteristics of PQQ-dependent ADH in acetic acid bacteria. Appl. Microbiol. Biotechnol. 70, 366–373. doi: 10.1007/s00253-005-0073-z, PMID: 16133326

[ref54] Treu B. L. Arechederra R. Minteer S. D. (2009). Bioelectrocatalysis of Ethanol via PQQ-Dependent Dehydrogenases Utilizing Carbon Nanomaterial Supports. J. Nanosci. Nanotechnol. 9, 2374–2380. doi: 10.1166/jnn.2009.SE33 19437978

[ref55] Vekeman B. Speth D. Wille J. Cremers G. de Vos P. op den Camp H. J. M.. (2016). Genome characteristics of two novel type I Methanotrophs enriched from North Sea sediments containing exclusively a lanthanide-dependent XoxF5-type methanol dehydrogenase. Microb. Ecol. 72, 503–509. doi: 10.1007/s00248-016-0808-7, PMID: 27457652

[ref56] Vu H. N. Subuyuj G. A. Vijayakumar S. Good N. M. Martinez-Gomez N. C. Skovran E. (2016). Lanthanide-dependent regulation of methanol oxidation Systems in *Methylobacterium extorquens* AM1 and their contribution to methanol growth. J. Bacteriol. 198, 1250–1259. doi: 10.1128/jb.00937-15, PMID: 26833413 PMC4859578

[ref57] Wehrmann M. Billard P. Martin-Meriadec A. Zegeye A. Klebensberger J. (2017). Functional role of lanthanides in enzymatic activity and transcriptional regulation of Pyrroloquinoline Quinone-dependent alcohol dehydrogenases in *Pseudomonas putida* KT2440. MBio 8, e00570–e00517. doi: 10.1128/mBio.00570-17 28655819 PMC5487730

[ref58] Yakushi T. Matsushita K. (2010). Alcohol dehydrogenase of acetic acid bacteria: structure, mode of action, and applications in biotechnology. Appl. Microbiol. Biotechnol. 86, 1257–1265. doi: 10.1007/s00253-010-2529-z, PMID: 20306188

[ref59] Yuhashi N. Tomiyama M. Okuda J. Igarashi S. Ikebukuro K. Sode K. (2005). Development of a novel glucose enzyme fuel cell system employing protein engineered PQQ glucose dehydrogenase. Biosens. Bioelectron. 20, 2145–2150. doi: 10.1016/j.bios.2004.08.017, PMID: 15741089

[ref60] Zelezniak A. Andrejev S. Ponomarova O. Mende D. R. Bork P. Patil K. R. (2015). Metabolic dependencies drive species co-occurrence in diverse microbial communities. Proc. Natl. Acad. Sci. 112, 6449–6454. doi: 10.1073/pnas.1421834112, PMID: 25941371 PMC4443341

[ref61] Zhai X. Wu K. Ji R. Zhao Y. Lu J. Yu Z.. (2022). Structure and function insight of the α-glucosidase QsGH13 from Qipengyuania seohaensis sp. SW-135. Front. Microbiol. 13:849585. doi: 10.3389/fmicb.2022.849585, PMID: 35308395 PMC8928221

[ref62] Zulic Z. Minteer S. D. (2011). Induced evolution of PQQ-dependent alcohol dehydrogenase activity in Gluconobacter sp.33 for use in enzymatic biofuel cells. J. Biobaased Mater. Bioenergy 5, 63–69. doi: 10.1166/jbmb.2011.1120

