# Supplementary material for: Enzymatic properties of alcohol dehydrogenase PedE_M.s. derived from Methylopila sp. M107 and its broad metal selectivity
Source: Front Microbiol. 2023 Jul 25;14:1191436. doi: 10.3389/fmicb.2023.1191436 (PMC10409515; doi:10.3389/fmicb.2023.1191436)
Supplement: Supplementary file 1 [file Data_Sheet_1.docx]

***Supplementary Material***


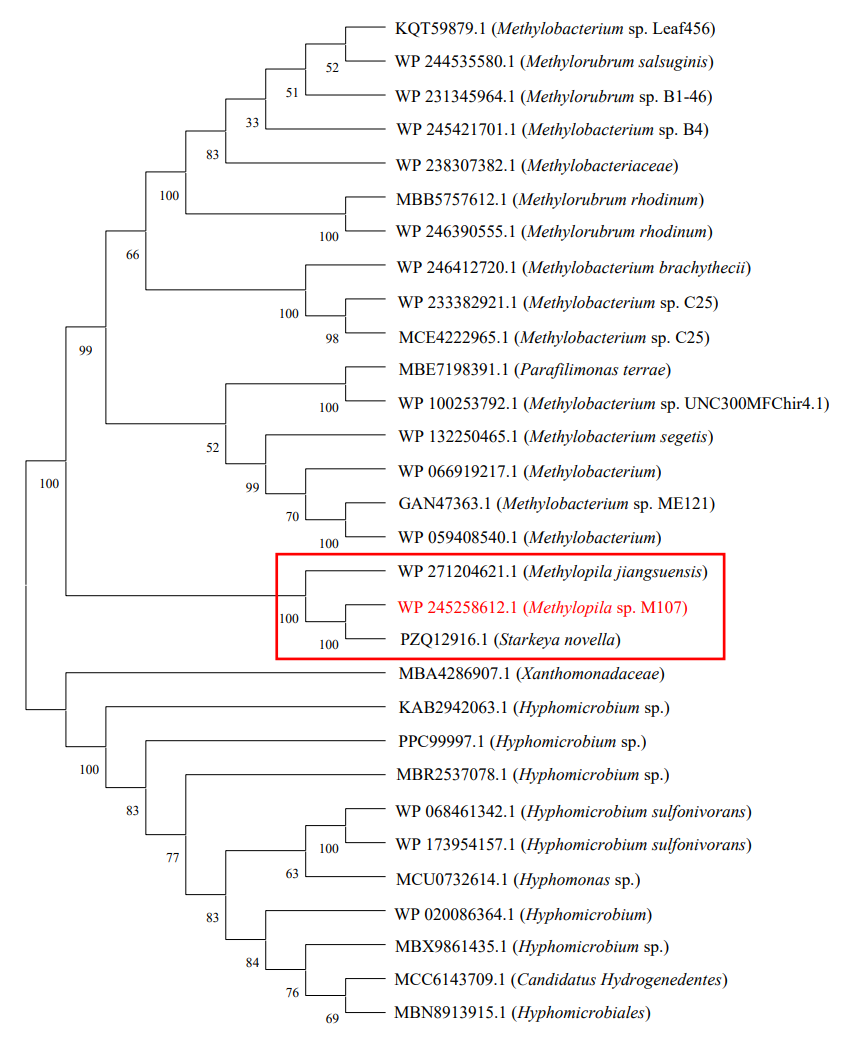


**Fig. S1** BLAST was used for sequence alignment, and sequences with the top 30 similarity beats were selected for phylogenetic tree analysis. The phylogenetic tree was obtained by MEGA X using the neighbor-joining (NJ) method. The numbers at the end of the phylogenetic tree denote the accession number, while the numbers on the branches indicate the level of confidence. Greater confidence values indicate a higher degree of similarity between the sequences. The strain origins of accession numbers were indicated in parentheses. The target protein in this study was highlighted with red characters, and the proteins within the red box are all PQQ-dependent PedE-type ADHs.


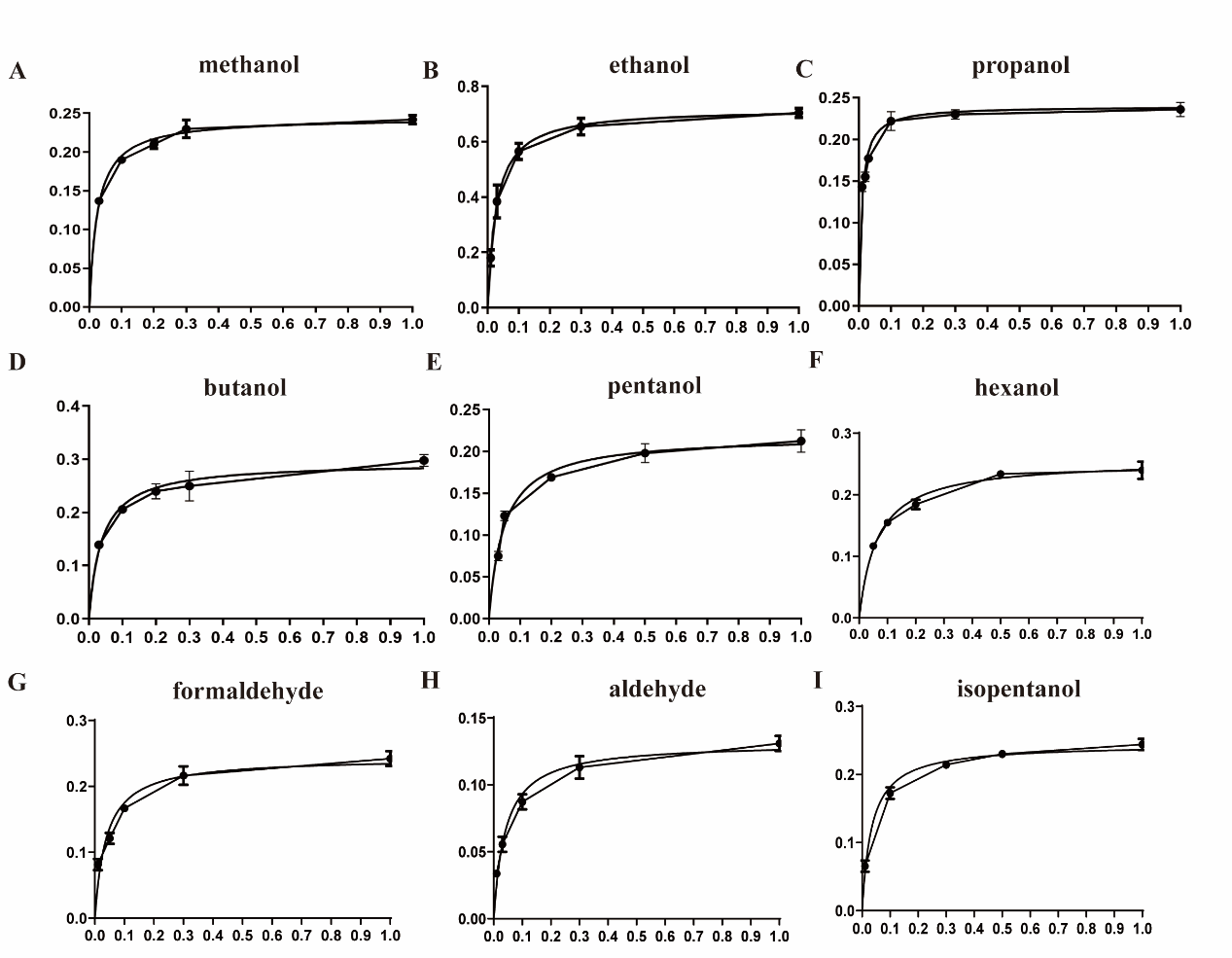


**Fig. S2** A to I indicate the kinetic curves for methanol, ethanol, propanol, butanol, pentanol, hexanol, formaldehyde, aldehyde and isopropanol, respectively; the horizontal coordinate indicates the substrate concentration (mM) and the vertical coordinate indicates the enzyme activity (U/mg). All data were measured by three biologically independent experiments. The error bars are shown in the figure.

**TABLE S1 |** Growth of bacteria on plates with different substrates.

|  | Methanol | ethanol | propanol | butanol | pentanol | hexanol | formaldehyde | aldehyde | | isopropanol |
| --- | --- | --- | --- | --- | --- | --- | --- | --- | --- | --- |
| La^3+^ | + | + | + | + | + | + | - | + | + | |
| Ca^2+^ | +++ | +++ | +++ | ++ | ++ | + | - | ++ | ++ | |

More "+" means better growth. All results were obtained through three biologically independent experiments.

**TABLE S2 |** Comparison of *K*_m_ value of ethanol by different PedE enzymes.

| **Enzyme** | **Organism** | ***K*_m_ (mM)** | | **Reference** | | |
| --- | --- | --- | --- | --- | --- | --- |
| PedE_M.s. | *Methylopila* sp. M107 | | 0.028±0.006 | | | This study |
| PedE_P.p. | *Pseudomonas putida* KT2440 | | 0.085±0.018 | | | (Wehrmann et al., 2017) |
| PedE_DSM3504 | *Gluconobacter* sp. 33 | | 0.14±0.08 | | | (Aquino Neto et al., 2015) |
| GsADH | *Geobacillus stearothermophilus* DSM 2334 | | 2.95 | | (Guo et al., 2019) | |
| FaADH | *Frateuria aurantia* LMG 1558T | | 11.76 | | (Trček and Matsushita, 2013) | |

REFERENCES

Aquino Neto, S., Hickey, D. P., Milton, R. D., De Andrade, A. R., and Minteer, S. D. (2015). High current density PQQ-dependent alcohol and aldehyde dehydrogenase bioanodes. *Biosensors and Bioelectronics* 72, 247–254. doi: 10.1016/j.bios.2015.05.011.

Guo, X., Feng, Y., Wang, X., Liu, Y., Liu, W., Li, Q., et al. (2019). Characterization of the substrate scope of an alcohol dehydrogenase commonly used as methanol dehydrogenase. *Bioorg Med Chem Lett* 29, 1446–1449. doi: 10.1016/j.bmcl.2019.04.025.

Trček, J., and Matsushita, K. (2013). A unique enzyme of acetic acid bacteria, PQQ-dependent alcohol dehydrogenase, is also present in Frateuria aurantia. *Appl Microbiol Biotechnol* 97, 7369–7376. doi: 10.1007/s00253-013-5007-6.

Wehrmann, M., Billard, P., Martin-Meriadec, A., Zegeye, A., and Klebensberger, J. (2017). Functional Role of Lanthanides in Enzymatic Activity and Transcriptional Regulation of Pyrroloquinoline Quinone-Dependent Alcohol Dehydrogenases in *Pseudomonas putida* KT2440. *mBio* 8, e00570-17. doi: 10.1128/mBio.00570-17.
